# Impact of Hemodialysis Catheter Dysfunction on Dialysis and Other Medical Services: An Observational Cohort Study

**DOI:** 10.1155/2012/673954

**Published:** 2012-02-12

**Authors:** Robert I. Griffiths, Britt B. Newsome, Grace Leung, Geoffrey A. Block, Robert J. Herbert, Mark D. Danese

**Affiliations:** ^1^Department of Epidemiology, Outcomes Insights, Inc., Westlake Village, CA 91362, USA; ^2^Division of General Internal Medicine, Johns Hopkins University School of Medicine, Baltimore, MD 21205, USA; ^3^Department of Clinical Research, Denver Nephrologists, P.C., Denver, CO 80218, USA; ^4^Department of Health Economics, Genentech, Inc., South San Francisco, CA 94080, USA; ^5^Department of Health Policy and Management, Johns Hopkins Bloomberg School of Public Health, Baltimore, MD 21205, USA

## Abstract

Practice guidelines define hemodialysis catheter dysfunction as blood flow rate (BFR) <300 mL/min. We conducted a study using data from DaVita and the United States Renal Data System to evaluate the impact of catheter dysfunction on dialysis and other medical services. Patients were included if they had ≥8 consecutive weeks of catheter dialysis between 8/2004 and 12/2006. Actual BFR <300 mL/min despite planned BFR ≥300 mL/min was used to define catheter dysfunction during each dialysis session. Among 9,707 patients, the average age was 62,53% were female, and 40% were black. The median duration of catheter dialysis was 190 days, and the cohort accounted for 1,075,701 catheter dialysis sessions. There were 70,361 sessions with catheter dysfunction, and 6,33 1 (65.2%) patients had at least one session with catheter dysfunction. In multivariate repeated measures analysis, catheter dysfunction was associated with increased odds of missing a dialysis session due to access problems (Odds ratio [OR] 2.50; *P* < 0.001), having an access-related procedure (OR 2.10; *P* < 0.001), and being hospitalized (OR 1.10; *P* = 0.001). Catheter dysfunction defined according to NKF vascular access guidelines results in disruptions of dialysis treatment and increased use of other medical services.

## 1. Background

Blood flow rate (BFR) <300 mL/min often is used to define hemodialysis catheter dysfunction, including in the National Kidney Foundation's (NKF) Kidney Disease Outcomes Quality Initiative (KDOQI) vascular access guidelines [1, 2], and in many research studies [3]. Causes of catheter dysfunction include mechanical kinking, malpositioning of the catheter tip, thrombus accumulation, and growth of a fibrin sheath [4]. Early dysfunction, which has been defined as occurring within the first two weeks of placement [2], is most often, but not exclusively, caused by mechanical problems. Delayed or late dysfunction is typically caused by thrombus accumulation, with or without the presence of a fibrin sheath [4], and is considered to be the most likely cause of low BFR overall [4–7].

Other definitions of catheter dysfunction reported in the literature include frequent arterial and venous pressure alarms, poor conductance, and poor dialysis efficiency based on urea reduction ratio or Kt/V calculations [4]: these have been applied when evaluating the impact of catheter dysfunction on clinical outcomes, economic expenditures, and patient quality of life [8–11]. However, the impact of catheter dysfunction using a BFR threshold, such as in the NKF-KDOQI guidelines, has received less attention. One notable exception is a recent study examining the relationship between hemodialysis catheter BFR and dialysis adequacy in a cohort of 259 patients at two university-based centers [12]. The premise for this study was that since the NKF-KDOQI blood flow threshold for catheter dysfunction was opinion based [1] and has been interpreted to mean that maintaining BFR > 300 mL/min is necessary for adequate dialysis, it is important to better understand the association between BFR and dialysis adequacy. The study found that mean BFRs < 300 mL/min were not commonly associated with dialysis inadequacy, leading the authors to conclude that strict adherence to the guideline could result in a significant number of unnecessary interventions. To our knowledge, however, the impact of hemodialysis catheter BFR <300 mL/min on dialysis and other medical services has not been evaluated.

The objective of this study was to identify medical service utilization, including missed sessions, access-related procedures, and all-cause hospitalizations, associated with dialysis catheter dysfunction defined according to a BFR threshold <300 mL/min.

## 2. Methods

### 2.1. Study Design and Setting

We conducted a retrospective cohort study using data from DaVita Inc. and the United States Renal Data System (USRDS). DaVita serves approximately 110,000 dialysis patients throughout the USA. The DaVita clinical data warehouse is a repository for detailed demographic, treatment, medication, and laboratory information. Information is available for each patient's individual dialysis sessions, allowing the investigator to reconstruct detailed longitudinal treatment histories.

 The USRDS is a national data system that collects, analyzes, and distributes information about end-stage renal disease (ESRD) [13]. The dataset includes the Centers for Medicare and Medicaid Services' (CMS), Renal Beneficiary and Utilization System (REBUS), and the ESRD Standard Analysis Files (SAF). REBUS contains demographic, diagnosis, and treatment history information for all Medicare beneficiaries with ESRD, a point prevalent cohort of approximately 570,000 in the second quarter of 2009 [14]. The SAFs contain 100% of Part A and Part B institutional claims and Part B physician supplier claims for these patients. The dataset used in this project consisted of a point prevalent dialysis patient population in August 2004, with a maximum followup period through December 31, 2006.

### 2.2. Participants

**Figure 1:**
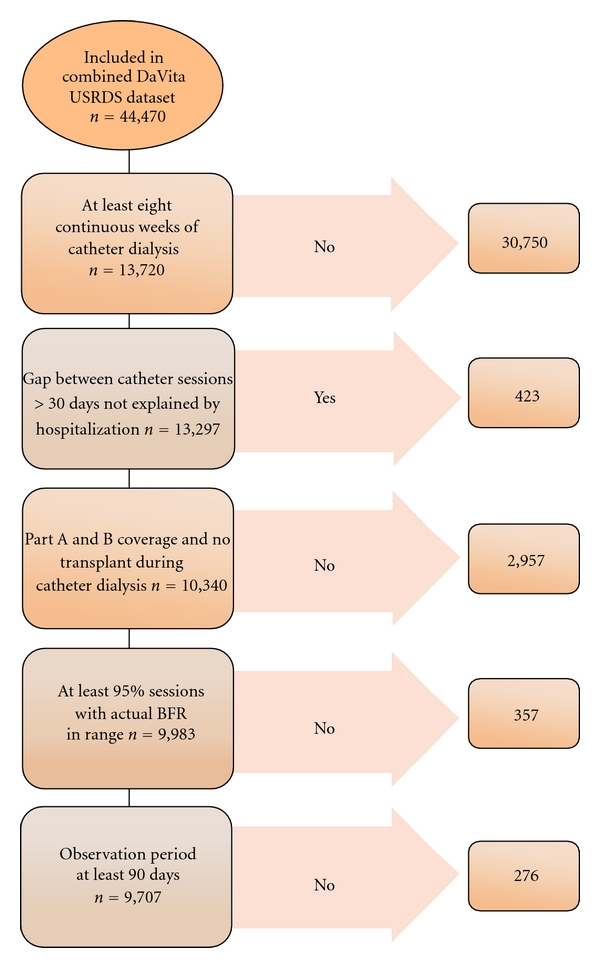
Patient inclusion and exclusion criteria.

Patients were included in this study based on the following criteria: they had at least eight continuous weeks of hemodialysis exclusively through a catheter between August 1, 2004, and December 31, 2006; in the first eight weeks of catheter dialysis, they did not have a gap between two consecutive outpatient dialysis sessions >30 days in which they were not hospitalized; they had both Part A and Part B Medicare coverage during the entire catheter dialysis period; they did not have a kidney transplant during the entire catheter dialysis period; at least 95% of their catheter dialysis sessions had actual and planned BFRs between 100 mL/min and 500 mL/min; they were alive and in the dataset for at least 90 days following the first catheter dialysis session (Figure 1). Planned and actual BFR values <100 mL/min or >500 mL/min were set to missing to minimize the potential impact of coding errors. In the final cohort, 99.9% of BFR values were within this range. Patients were followed from their first catheter dialysis session (defined as their index date), to their last catheter dialysis session that was uninterrupted by either a change in access or dialysis modality. This was defined as their observation period.

### 2.3. Variables

 For each patient included in the study, we reconstructed a longitudinal history of catheter dialysis and medical resource use during their observation period. Reasons for reaching the end of the observation period were (i) death, if the patient died on or before December 31, 2006, and if the last catheter dialysis session was within 30 days of death, (ii) end of data (censored), if the last catheter dialysis session was within 30 days of December 31, 2006, or (iii) change in access type or modality, if the last observed catheter dialysis session was not due to either death or the end of data.

Catheter dysfunction was defined as actual BFR <300 mL/min despite a planned BFR ≥300 mL/min. A slight modification of the NKF-KDOQI vascular access guideline was adopted to eliminate misclassification of catheter dysfunction where the intent, as indicated by planned BFR, was to provide BFR <300 mL/min. The outcome variables in this study were dialysis run time (in minutes), missed dialysis session due to access problems, access-related procedures, and all-cause hospitalization. The DaVita data contained a record for each missed session, which included the date and the reason for the missed session, including “access problems.” Access-related procedures were identified using Health Care Common Procedure Coding System (HCPCS) codes from Medicare claims. The “limited” definition of access-related procedures consisted of the following: injection for catheter evaluation with fluoroscopy (36598); thrombolytic declotting of catheter (36593); mechanical removal of clot (36596); Mechanical removal of intraluminal (intracatheter) obstructive material (75902); injection of “TPA” (J2997). The “expanded” definition also included tunneled catheter exchange or replacement (36581) and the combination of removal of tunneled catheter (36589) *plus* tunneled catheter insertion (36558). Hospitalizations consisted of all acute care admissions for any reason.

### 2.4. Analyses

**Figure 2:**
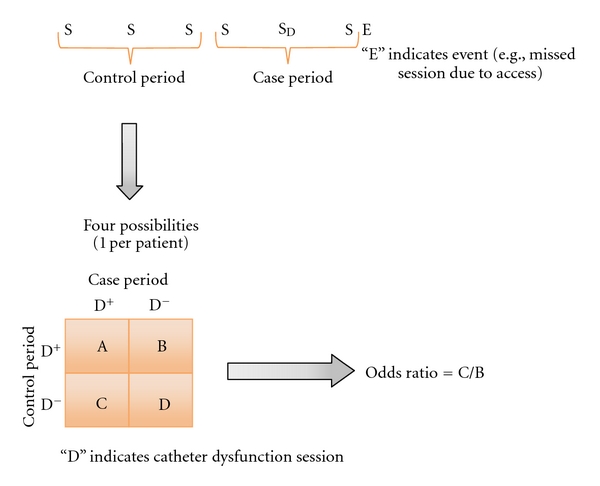
Case-crossover design.

We used two different approaches to analyze associations between catheter dysfunction sessions and economic outcomes: case-crossover [15] and multivariate-repeated measures analysis. In the case-crossover analyses (Figure 2), we included only patients who had the event of interest (e.g., missed dialysis session due to access problem). For each of these patients, we identified the first event at least six sessions following the beginning of catheter dialysis. As shown in Figure 2, we divided the six dialysis sessions immediately preceding the event into two exposure periods: the case period was defined as sessions 1–3 immediately preceding the event, and the control period was defined as sessions 4–6 preceding the event. For each patient, we identified the presence of at least one session with catheter dysfunction within each of the two periods, case and control. Patients were then divided into four groups, as illustrated in the two-by-two table within Figure 2: catheter dysfunction in both periods (labeled “A” in the two-by-two table); no catheter dysfunction in either period (D); catheter dysfunction in the case but not the control period (C); catheter dysfunction in the control but not the case period (B). The odds of the exposure (in this case, the presence of at least one catheter dysfunction session) being associated with the outcome of interest is defined as the ratio of the count of patients with a catheter dysfunction session in the case, but not the control, period divided by the count of patients with a catheter dysfunction session in the control, but not the case, period (C/B in Figure 2). As patients serve as their own controls, there is no need to adjust this ratio for covariables. We conducted a sensitivity analysis, where we changed the lengths of the case and control periods from three sessions each to one session each.

Multivariate-repeated measures analyses [16, 17] were used as the second approach. All patients in the catheter dialysis cohort were included in these analyses, as were all of their sessions. These analyses used individual dialysis sessions as the repeated measures and incorporated as covariables patient age, gender, race, underlying cause of renal failure, ESRD network, dialysis vintage, Charlson Comorbidity Index [18], days since the start of catheter dialysis, and whether the patient could have started catheter dialysis prior to the beginning of the observation window. Each dialysis session included a binomial outcome variable indicating whether the outcome, for example, missed session due to access problems, occurred between the date of the current session and the date of the following session. Therefore, the repeated measures analysis was designed to measure the relative odds of the outcome occurring before the following dialysis session, among those with versus without catheter dysfunction in the previous session. In addition to specifying catheter dysfunction as a dichotomous variable (BFR < 300 mL/min versus ≥300 mL/min, the reference category), we created five intervals of BFR (100–<150 mL/min; 150–<200 mL/min; 200–<250 mL/min; 250–<300 mL/min; and ≥300 mL/min, the reference category) and then repeated all the multivariate analyses using this specification instead of the dichotomous specification as the independent variable for catheter dysfunction. In these analyses, we also included dialysis run time in minutes as an independent variable. Analysis file construction, descriptive analyses, and the case-crossover analyses were performed in SAS (version 9.1.3) [19]. The repeated measures analyses were performed in STATA (version 10) [20]. 

## 3. Results

Of 44,470 patients in the combined DaVita USRDS database, 9,707 (22%) met all the inclusion criteria (Figure 1). The average age of the cohort was 62 years (range 18–102), 53% were female gender, 40% were black race, 46% had diabetes, 28% had hypertension recorded as their underlying cause of renal failure, and 63% had a Charlson Comorbidity Index of ≤2 (Table 1). The average duration of ESRD prior to entering the cohort was 40 months (range 0–367).

The median duration of catheter dialysis was 190 days (Interquartile range 108–386 days). Reasons for the end of catheter dialysis were as follows: 7,476 (77%) switched to another dialysis access type or modality and 1,068 (11%) died while receiving catheter dialysis. There were 1,163 (12%) patients who were alive and on catheter dialysis at the end of the data (12/31/06).

The cohort accounted for 1,075,701 catheter dialysis sessions over the entire observation period (mean 111; median 73; range 7–502) and 218,166 sessions during the first eight weeks of catheter dialysis (mean 22; median 24; range 3–48; mean per week 3 sessions). There were 1,074,966 (99.9% of 1,075,701) sessions with a planned BFR between 100 and 500 mL/min: 400 mL/min (38%); 350 mL/min (33%); 300 mL/min (13%); <300 mL/min (1%). In contrast to planned BFR, 7% (77,628) had an actual BFR <300 mL/min. There were 70,361 sessions with actual BFR <300 mL/min despite planned BFR ≥300 mL/min (catheter dysfunction) and 6,331 (65.2%) patients had at least one session with catheter dysfunction (mean = 11.1 sessions; median = 5 sessions).

In the baseline case-crossover analysis (Table 2), using three case sessions and three control sessions, catheter dysfunction was associated with increased odds of a missed session due to access problems, an access-related procedure (both limited and expanded definition), and either a missed session due to access problems or an access-related procedure. Catheter dysfunction was associated with increased odds of all-cause hospitalization in the sensitivity analysis using one case and one control session, but not in the baseline analysis.

In the multivariate-repeated measures analysis that included catheter dysfunction specified as a dichotomous variable (BFR < 300 mL/min versus ≥300 mL/min, the reference category, Table 3), BFR <300 mL/min was associated with increased odds of a missed session due to access problems, access-related procedure (limited and expanded definition), missed session or access-related procedure, and all-cause hospitalization (odds ratio 1.10; *P* = 0.001). The association between catheter dysfunction and dialysis run time in the same session was not significant.

In the multivariate-repeated measures analysis that included five intervals of BFR (100–<150 mL/min; 150–<200 mL/min; 200–<250 mL/min; 250–<300 mL/min; ≥300 mL/min, the reference category) and included dialysis run time in minutes as an independent variable (Table 4), lower BFR was associated with increased odds of all the outcomes. With the exception of the odds ratio for hospitalization at BFR 250–<300 mL/min, all the coefficients were statistically significant (*P* ≤ 0.05).

## 4. Discussion

Using DaVita and USRDS data, we were able to identify a large cohort of almost 10,000 dialysis patients who received exclusively catheter dialysis for at least eight weeks between August 2004 and December 2006. The median duration of catheter dialysis was longer than 26 weeks, and most patients switched to a different type of access or modality before December 2006, so we were able to document precisely the end of uninterrupted catheter use. The proportion of patients receiving catheter dialysis in this study is consistent with current point prevalent estimates of chronic catheter use in the dialysis population overall.

The patients in our cohort accounted for more than one million catheter dialysis sessions. We found that approximately 70,000 sessions, or 7% of those with both a planned and an actual BFR value present, had an actual BFR <300 mL/min despite a planned BFR ≥300 mL/min, a definition of catheter dysfunction that closely approximates NKF-KDOQI vascular access guidelines. This illustrates that catheter dysfunction defined according to the BFR threshold is a common problem, affecting approximately one in 14 sessions or one patient session every month. Almost two thirds of all patients had at least one session with catheter dysfunction, and more than 25% of these had at least 12 such sessions, suggesting that catheter dysfunction may be an ongoing problem in some patients.

Using two different analytic approaches, case-crossover and multivariate-repeated measures analysis, we found strong associations between the presence of catheter dysfunction and increased risk of a missed session due to access problems, access-related procedures, a missed session due to access problems or access procedure, and all-cause hospitalization. In multivariate-repeated measures analysis, when we changed the specification of the catheter dysfunction independent variable from dichotomous (BFR < 300 mL/min versus ≥300 mL/min) to one that included multiple levels (100–<150 mL/min; 150–<200 mL/min; 200–<250 mL/min; 250–<300 mL/min; ≥300 mL/min) and included dialysis run time in minutes as an additional independent variable, we found strong associations between the level of catheter dysfunction and the odds of the outcome.

 Each of these analytic approaches has strengths and limitations. In the case-crossover approach, we included only patients who had the outcome of interest (e.g., missed session due to access problems). Further, we considered only the first such event for each patient, ignoring those that may have occurred later in the patient's catheter dialysis history. One important advantage of the case-crossover approach is that patients serve as their own controls because the risk of “exposure,” in this instance catheter dysfunction in at least one of three sessions before the event, is compared during two different periods for each individual patient. Consequently, there is no need to adjust for many of the factors that can confound associations between exposure and outcomes. Case-crossover designs are not immune to problems of confounding, however, as temporal changes within patients can confound comparisons between the control and the case period. We do not believe this was a significant issue in our study since the case and control periods were limited to a maximum of three dialysis sessions each.

In the multivariate-repeated measures approach, we included all patients, all catheter sessions, including those defined as having dysfunction, and all outcomes events of interest during the observation period. One limitation of this approach is that since it includes both patients with and without at least one session with catheter dysfunction, it is necessary to adjust for differences between patients that may confound observed associations between catheter dysfunction and medical services. It is possible, therefore, that observed differences in patterns of medical services between those with versus without catheter dysfunction may reflect unobserved differences in patient characteristics.

Our study has several other limitations. First, our data source, as in most retrospective analyses, lacked some clinical variables that would have strengthened the study. For example, although we sought to characterize catheter dysfunction according to the most recent NKF-KDOQI guidelines as “failure to attain and maintain an extracorporeal blood flow of 300 mL/min or greater at a prepump arterial pressure more negative than −250 mm Hg,” we did not have access to information on prepump arterial pressure or whether or not the patients had undergone line reversal in our dataset. Although this limited our ability to exactly replicate the NKF-KDOQI criteria, the inclusion of blood flow represents a significant clinical focus in assessing a catheter's ability to provide an adequate dialysis treatment. Additionally, we were unable to specifically identify the brand of catheter, the precise handling techniques, or the use of locking solution for each catheter session although presumably most dialysis sessions would have been conducted under standardized guidelines as described by DaVita clinical policies and procedures. Another limitation of the data is that we did not have access to a variable indicating dialysis center and included dialysis Network in lieu of center. Also, to limit false-positive catheter dysfunction, we restricted our definition to those sessions with actual BFR <300 mL/min and planned BFR ≥300 mL/min, and we set to missing actual and planned BFR values <100 mL/min. As a result, we have almost certainly underestimated the number of sessions with catheter dysfunction, and possibly also the effect of catheter dysfunction on utilization of medical services. These differences may, in part, account for the discrepancy between the proportion of sessions with catheter dysfunction in this study and the proportion previously reported by CMS [21].

Second, by requiring patients to have at least eight weeks of catheter dialysis, and to have survived at least 90 days following the start of dialysis, we have excluded patients who were on catheter dialysis for shorter periods of time or who died within 90 days of beginning catheter dialysis. To the extent that catheter dysfunction is more common sooner after placement, by excluding patients with short-term catheter dialysis, we may have underestimated the overall rate of dysfunction. Catheter dysfunction due to mechanical reasons, which is known to occur sooner rather than later after placement, may be disproportionately underrepresented. Also, if death during the first 90 days after catheter placement is related to serious complications of catheter dysfunction, such as bloodstream infection, by requiring at least 90 days' survival, we may have underestimated the impact of catheter dysfunction on the use of medical services, in particular, on all-cause hospitalization.

Third, our study population consisted of a point prevalent cohort of patients who were receiving dialysis services at DaVita in August 2004, and who, by definition, had their first ESRD service before or during that month. We did not have access to detailed dialysis session data, including type of dialysis, prior to August 2004. Consequently, we could not determine the actual start of catheter dialysis for those who had their first ESRD service before the beginning of the DaVita data and their first documented catheter dialysis session in August 2004. It is highly likely, however, that some of these patients began catheter dialysis months before entering our cohort. Again, to the extent that catheter dysfunction occurs sooner rather than later following placement, had we been able to observe all patients from the start of catheter dialysis, we may have observed a higher rate of dysfunction, and different patterns of medical services.

Finally, we were unable to determine whether the disruptions to dialysis services and the utilization of other medical services we observed were necessary to ensure adequate dialysis or an unnecessary response based only on reaching the BFR threshold in the vascular access guidelines. Dialysis facilities have strong financial incentives to operate at full capacity, and any missed dialysis session may represent a loss of revenue for the facility. Also, it seems unlikely that low BFR alone would be sufficient to result in hospitalization. However, it is more difficult to draw conclusions regarding other medical services observed in this study, especially in light of the study by Moist and colleagues that showed BFR <300 mL/min is not an accurate predictor of dialysis inadequacy [12].

## 5. Conclusions

In spite of these limitations, which we believe may have resulted in underestimating both the rate of catheter dysfunction and the strength of the associations between catheter dysfunction and use of medical services, we found that catheter dysfunction defined according to NKF-KDOQI is common and results in disruptions in the provision of dialysis services and utilization of additional medical services. Efforts should continue to reduce chronic catheter use, and to minimize the clinical and economic consequences of catheter dysfunction through early detection and intervention. Further research would be required to determine the direct impact of the vascular access guideline on unnecessary procedures. Our study should be repeated in other countries, where the epidemiology and outcomes of catheter dysfunction may differ from the USA. Also, conducting this study in patients with arteriovenous fistula or graft would help validate our findings on inadequate BFR and outcomes.

## Figures and Tables

**Table 1:** Demographic and clinical characteristics of the study population.

Characteristic	Frequency	%
	9,707	100.0%
Age		
18–49	2,080	21.43%
50–64	3,073	31.66%
65–74	2,278	23.47%
≥75	2,276	23.45%
Gender		
Male	4,587	47.3%
Female	5,120	52.7%
Race		
Black	3,903	40.2%
White	5,158	53.1%
Other	646	6.7%
Underlying cause of renal failure		
Diabetes	4,456	45.9%
Hypertension	2,678	27.6%
Glomerulonephritis	977	10.1%
Other/unknown	1,596	16.4%
Charlson comorbidity index score distribution		
0	4,038	41.6%
1-2	2,068	21.3%
3-4	2,058	21.2%
≥5	1,543	15.90%
ESRD network*		
01-02 (CT, MA, ME, NH, NY, RI, VT)	458	4.7%
03-04 (DE, NJ, PA)	416	4.3%
05 (DC, MD, VA, WV)	1,018	10.5%
06 & 08 (AL, GA, MS, NC, SC, TN)	1,222	12.6%
07 (FL)	789	8.1%
09-10 (IL, IN, KY, OH)	787	8.1%
11 (MI, MN, ND, SD, WI)	763	7.9%
12 (IA, KS, MO, NE)	375	3.9%
13 (AR, LA, OK)	392	4.0%
14 (TX)	1,095	11.3%
15 (AZ, CO, NM, NV, UT, WY)	581	6.0%
16-17 (AK, CA, HI, ID, MT, NoCA, OR, WA)	824	8.5%
18 (SoCal)	987	10.2%

*Abbreviations are US states within each region.

**Table 2:** Case-crossover analysis of medical services associated with catheter dysfunction.

Type of medical service	3 Case* sessions	3 Control** sessions	Odds ratio (*P*-value)	1 Case session	1 Control session	Odds ratio (*P*-value)
Missed session due to access problems	103	66	1.56 (<0.01)	94	56	1.68 (<0.01)
Access procedure						
Limited definition***	301	234	1.29 (<0.01)	212	179	1.18 (0.10)
Expanded definition****	365	290	1.26 (<0.01)	289	226	1.28 (<0.01)
Missed session or access procedure	398	291	1.37 (<0.0001)	321	246	1.30 (<0.01)
Hospitalization (any diagnosis)	395	354	1.12 (0.13)	293	225	1.30 (<0.01)

*Sessions immediately prior to event, that is, sessions 1–3 prior to event.

**Sessions before case sessions, that is, sessions 4–6 prior to event.

***Consists of injection for catheter evaluation with fluoroscopy (36598); thrombolytic declotting of catheter (36593); mechanical removal of clot (36596); mechanical removal of intraluminal (intracatheter) obstructive material (75902); injection of “TPA” (J2997).

****Consists of those procedures included in the limited definition, plus tunneled catheter exchange or replacement (36581), and the combination of removal of tunneled catheter (36589) *plus* tunneled catheter insertion (36558).

**Table 3:** Repeated measures analysis* of medical services associated with catheter dysfunction.

Type of medical service	Odds ratio**	95% confidence interval		*P*-value
		Lower	Upper	
Missed session due to access problems	2.50	2.10	2.97	<0.001
Access procedure				
Limited definition***	2.10	1.97	2.25	<0.001
Expanded definition****	1.17	1.10	1.25	<0.001
Missed session or access procedure	2.21	2.11	2.33	<0.001
Hospitalization (any diagnosis)	1.10	1.04	1.17	0.001

*Multivariate analyses included age, gender, race, underlying cause of renal failure, dialysis vintage, ESRD Network, Charlson Comorbidity Index, and whether the patient was incident to catheter dialysis, as co-variables.

**Odds of medical service before the next dialysis session among those with versus without catheter dysfunction in the preceding session.

***Consists of injection for catheter evaluation with fluoroscopy (36598); thrombolytic de-clotting of catheter (36593); mechanical removal of clot (36596); mechanical removal of intraluminal (intracatheter) obstructive material (75902); injection of “TPA” (J2997).

****Consists of those procedures included in the limited definition, plus tunneled catheter exchange or replacement (36581), and the combination of removal of tunneled catheter (36589) *plus* tunneled catheter insertion (36558).

**Table 4:** Alternative specification of catheter dysfunction variable in repeated measures analysis* of medical services.

Type of medical service	Odds ratio**	95% confidence interval		*P*-value
		Lower	Upper	
Missed session due to access problems				
Blood flow rate ≥ 300 mL/min		Reference category		
100–<150 mL/min	8.09	3.75	17.45	<0.001
150–<200 mL/min	15.79	11.13	22.40	<0.001
200–<250 mL/min	3.14	2.46	4.02	<0.001
250–<300 mL/min	1.59	1.22	2.07	0.001
Access procedure				
Limited definition***				
Blood flow rate ≥ 300 mL/min		Reference category		
100–<150 mL/min	4.16	2.80	6.18	<0.001
150–<200 mL/min	4.14	3.38	5.08	<0.001
200–<250 mL/min	2.45	2.22	2.71	<0.001
250–<300 mL/min	1.78	1.63	1.93	<0.001
Expanded definition****				
Blood flow rate ≥ 300 mL/min		Reference category		
100–<150 mL/min	6.88	5.44	8.69	<0.001
150–<200 mL/min	5.63	4.88	6.49	<0.001
200–<250 mL/min	2.55	2.36	2.75	<0.001
250–<300 mL/min	1.72	1.61	1.83	<0.001
Missed session or access procedure				
Blood flow rate ≥ 300 mL/min		Reference category		
100–<150 mL/min	6.98	5.54	8.79	<0.001
150–<200 mL/min	5.81	5.05	6.69	<0.001
200–<250 mL/min	2.55	2.36	2.74	<0.001
250–<300 mL/min	1.72	1.61	1.83	<0.001
Hospitalization (any diagnosis)				
Blood flow rate ≥ 300 mL/min		Reference category		
100–<150 mL/min	1.59	1.01	2.51	0.05
150–<200 mL/min	1.49	1.18	1.87	0.001
200–<250 mL/min	1.24	1.13	1.37	<0.001
250–<300 mL/min	1.00	0.93	1.08	0.95

*Multivariate analyses included *dialysis run time (minutes)*, age, gender, race, underlying cause of renal failure, dialysis vintage, ESRD Network, Charlson Comorbidity Index, and whether the patient was incident to catheter dialysis, as covariables.

**Odds of medical service before the next dialysis session among those with versus without catheter dysfunction in the preceding session.

***Consists of injection for catheter evaluation with fluoroscopy (36598); thrombolytic declotting of catheter (36593); mechanical removal of clot (36596); mechanical removal of intraluminal (intracatheter) obstructive material (75902); injection of “TPA” (J2997).

****Consists of those procedures included in the limited definition, plus tunneled catheter exchange or replacement (36581), and the combination of removal of tunneled catheter (36589) *plus* tunneled catheter insertion (36558).
